# Enablers and Challenges in the Implementation of Active Case Findings in a Selected District of Karnataka, South India: A Qualitative Study

**DOI:** 10.1155/2020/9746329

**Published:** 2020-01-23

**Authors:** Amrita N. Shamanewadi, Poonam R. Naik, Pruthu Thekkur, Suwarna Madhukumar, Abhay Subhashrao Nirgude, M. B. Pavithra, Basavaraj Poojar, Vivek Sharma, Arnav Prashanth Urs, B. V. Nisarga, N. Shakila, Sharath Burugina Nagaraja

**Affiliations:** ^1^Department of Community Medicine, MVJ Medical College and Research Hospital (MVJMCRH), Dandupalya, Hoskote, Bengaluru Rural Pin-562114, India; ^2^Department of Community Medicine, Yenepoya Medical College, Yenepoya (Deemed to be University), Mangalore Pin-575018, India; ^3^Centre for Operational Research, International Union Against Tuberculosis and Lung Disease, Paris Pin-75006, France; ^4^Centre for Operational Research, The Union South-East Asia Office, New Delhi Pin-110016, India; ^5^Department of Pharmacology, Kasturba Medical College, Mangalore Pin-575003, India; ^6^Monitoring and Evaluation Division, THALI, JSI India, West Bengal Pin: 700107, India; ^7^District Tuberculosis Officer, Bengaluru Rural District, Bengaluru, India; ^8^Department of Community Medicine, ESIC Medical College and PGIMSR, Bengaluru Pin-560010, India

## Abstract

**Background:**

Active case finding (ACF) for tuberculosis (TB) is a promising tool to enhance early case detection among marginalized populations. As opposed to passive case finding, it involves systematically searching for TB in individuals who would not spontaneously present for care. The National TB Program (NTP) of India has initiated ACF for TB through the existing general health system since the end of 2017. However, prior to scale-up, there is need for exploring the implementation challenges and solutions to improve the efficiency of this program.

**Objectives:**

(1) To explore the enablers and challenges in the implementation of ACF for TB by NTP in the Bengaluru rural district of Karnataka, South India, and (2) to explore the perceived solutions to improve the efficiency of ACF activity.

**Methods:**

A qualitative descriptive study was conducted in the Bengaluru rural district during July 2018. In-depth interviews using purposively selected health care providers involved in active case finding (*n* = 9) and presumptive TB patients (*n* = 9) and presumptive TB patients (

**Results:**

The challenges in conduct of ACF were as follows: inadequate training of health care workers, shortage of staff, indifferent attitude of community due to stigma, lack of awareness about TB, illiteracy, inability to convince patients for sputum test, and delay in getting CBNAAT results. The field staff recommended the installation of mobile CBNAAT machine, involvement of general health staff in activity, training of health workers on counseling of patients, and issue of identity cards for community health workers/volunteers so that people recognize them.

**Conclusion:**

The health system challenges in conduct of ACF need to be addressed by training the health staff involved in activity and also improving the access to TB diagnostics.

## 1. Introduction

Globally, tuberculosis (TB) remains a major public health problem of concern, disproportionately affecting individuals in low- and middle-income countries (LMICs) [1]. In 2017, an estimated 10 million people developed TB and 1.3 million died due to TB. In the same year, about 6.7 million patients with TB were notified to National TB Programs (NTP), meaning that 3.3 million (33% of the estimated disease burden) either were not diagnosed or were diagnosed but not notified to NTP [2]. As failure to detect patients with TB and treat can lead to uninterrupted transmission of disease, the End TB Strategy of the World Health Organization (WHO) endorsed the early diagnosis of TB through strategies like active screening of high-risk groups [3].

India is a high TB burden country with the triple burden of TB, drug-resistant TB, and HIV-infected TB. India alone accounts for 27% of global TB burden, with an estimated 2.7 million new TB patients in the year 2017 [2]. However, only 1.9 million (30% of estimated) were not notified. One of the potential reasons for missing the TB patients in India was that the NTP relied heavily on a passive case finding strategy for diagnosis of TB [4]. With passive case finding, the diagnosis of TB was largely dependent on the health seeking behavior of the people with symptoms suggestive of TB. Acknowledging the deficiency of passive case finding and directives of End TB Strategy, the NTP implemented active case finding (ACF) among high-risk groups (HRGs) in 300 districts of India. Migrants and people living in urban slums, construction sites, and hard-to-reach areas were considered HRGs for the ACF activity. The NTP is planning to universally scale up the ACF and to conduct three rounds of ACF each year [4].

ACF is a novel and promising approach towards early TB case detection among high-risk and marginalized populations [5–7]. The studies have shown ACF to be beneficial in detecting the TB patients, reducing delay in diagnosis and also the out-of-pocket expenditure for diagnosis of TB. However, prompt linkage of individuals detected with symptoms for diagnosis and TB treatment is crucial to ACF's success, as failure to do so may result in disease progression and continued transmission within the community. Nevertheless, the studies elsewhere have shown deficiencies in the implementation of ACF, leading to attrition at various stages of the diagnosis and treatment. There is a similar anecdotal evidence on the lacunae in the ACF activity conducted by NTP, thus diminishing the benefits of the activity. The ACF activity of NTP was conducted with the existing NTP field staff and community health workers of the general health system.

The ACF activity was an additional activity over and above their routine health care service delivery. As NTP plans to scale up the ACF activity, insight on challenges faced by the health care providers during ACF activity and presumptive TB patients in adhering to the prescribed process of diagnosis can help in improving the efficiency of the activity. However, there is no literature on the enablers and challenges of ACF implementation as perceived by various stakeholders. Thus, we aimed to explore the perceptions of TB patients and health care providers on enablers and challenges in the implementation of active case finding in a selected district of Karnataka, South India. Also, we tried to explore the perceived solutions to improve the efficiency of ACF activity.

## 2. Methods

### 2.1. Study Design

This was a qualitative descriptive study.

### 2.2. Study Setting

The study was conducted in the Bengaluru rural district of the state of Karnataka, with a population of 66.8 million. Bengaluru rural district comprises four Talukas—Devanahalli (population: 209,622), Doddaballapura (population: 299,594), Hoskote (population: 270,818), and Nelamangala (population: 210,889). The district is mainly a plain area, with a total area of about 2298 km [2]. The population density of the district is 431 people per square kilometer. The district has four Tuberculosis Units (TU, a peripheral unit of NTP) with one each situated in the four Talukas of the district. There are 12 designated microscopy centers (DMCs) in the district, of which two are located in medical colleges, four in the subdistrict hospital, and six in primary health centers.

ACF is a unique effort made by NTP to reach the unreached in a campaign mode to improve TB case finding. In ACF conducted by NTP, systematic active screening was done in high-risk areas through house-to-house visits. Mapping of the high-risk areas and vulnerable population (people living in urban slums, mobile population and tribes, and people living in construction sites) was before the ACF activity. The ACF activity was conducted in preidentified high-risk areas.

A detailed microplan for ACF activity was prepared by a Medical Officer (MO) of a Tuberculosis Unit in consultation with District TB Officer (DTO). The frontline health workers of NTP, like Senior Treatment Supervisor (STS), Senior TB Laboratory Supervisor (STLS), and TB Health Visitor (TBHV), were involved in the activity. Also, the peripheral health workers from the general health system and an Accredited Social Health Activist (ASHA, a community volunteer) were included. For each identified high-risk area, one frontline health worker from either NTP or general health services was made in charge and tagged with either ASHA or any other community volunteers from the selected area. The two together had to cover about 500 houses over 2 to 3 days of activity.

During the ACF activity, the house visits were made to identify presumptive TB patients through symptom screening. Those with either cough for ≥2 weeks, fever for more than ≥2 weeks, significant weight loss (>5% weight loss for the last three months), or presence of blood in sputum any time during the last six months were considered presumptive TB patients. All identified presumptive TB patients were counseled for undergoing diagnostic tests to rule out TB. Post counseling, two sputum collection containers were provided for collecting spot and early morning sputum samples for sputum smear microscopy. Also, patients were briefed on procedures to produce, collect, and carry collected sputum samples to the designated microscopy center (DMC).

Sputum smear microscopy was done among all presumptive TB patients who submitted sputum samples to DMC. The people who were sputum positive were initiated on treatment. The presumptive TB cases who are sputum negative but symptomatic were further followed up and offered chest X-ray. Those who required CBNAAT as per RNTCP guidelines underwent Gene Xpert MTB/Rif assay [8].

**Figure 1:**
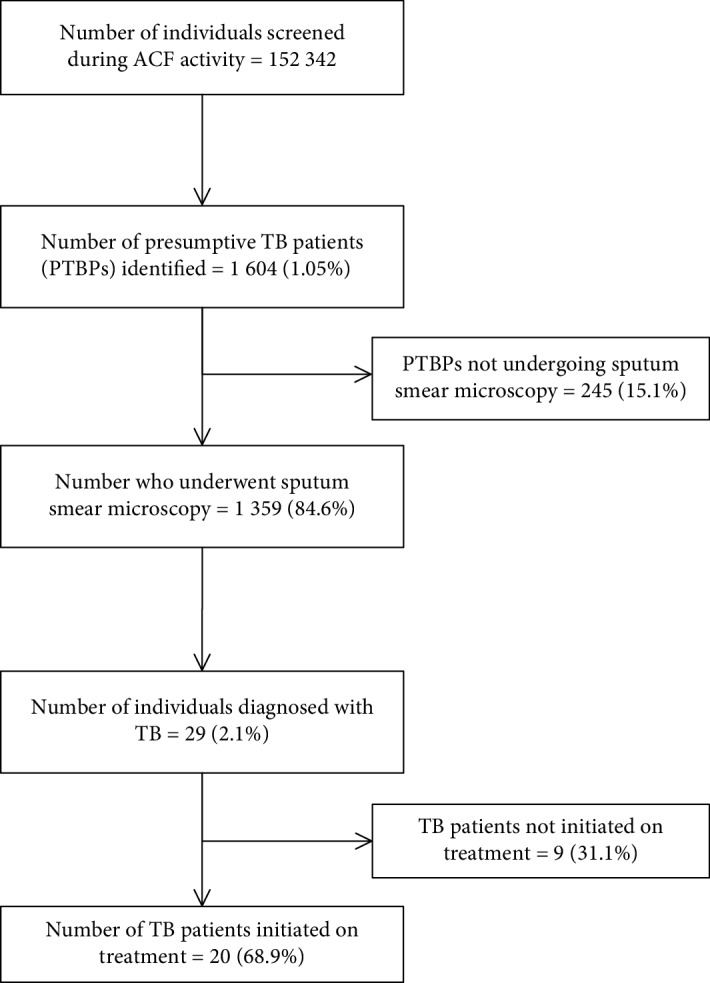
Number and percentage of participants at different stages of diagnostic and treatment cascade among presumptive TB patients identified by ACF activity in Bengaluru rural, Karnataka, India, during July 2018.

During the ACF activity round conducted during July 2018 in the Bengaluru rural district, a total of 152,342 individuals were screened. Of the screened, 1604 (1.1%) presumptive TB patients were identified, of which only 1359 (84.6%) underwent sputum smear microscopy. In total, 29 pulmonary TB patients were identified, of which 20 (68.9%) initiated on treatment (Figure 1). There was a low rate of identification of presumptive TB patients and high attrition during various stages of diagnosis and treatment cascade indicating potential deficiencies and challenges in the implementation of ACF activity.

### 2.3. Study Population

In total, eight presumptive TB patients (five male and three female patients) and nine health care providers were included in the study. We used a purposive sampling to select the health care providers with representation of different cadres of staff of TB program and the general health system. Two TBHVs, one STS, one laboratory technician working in a DMC, and five ASHAs involved in ACF activity of Bengaluru rural district were included. The presumptive TB cases identified in active case finding activity were conveniently selected and included. Written informed consent was taken from the participants before including them in the study.

### 2.4. Data Collection

In-depth interviews were conducted face-to-face in local language (Kannada) by the principal investigator (a female medical doctor (MD) trained in qualitative research) and audio recorded using a “voice recorder” mobile app after obtaining consent. The interviewer was working in a private medical college and was not involved in program implementation. Three participants did not give consent for audio recording, when field notes were taken. Separate interview schedules with probes were used to interview patients and health care providers. The interview schedule had questions and relevant probes to gather information on enablers and challenges for conducting ACF activity. The interviews of health care providers were conducted at their workplace. The patients' interview was conducted at their place of convenience (three at home, two at their workplace, and three in DMCs). Interviews lasted for an average of 29 minutes (range 20-45).

### 2.5. Data Analysis

Transcripts were prepared within a week of conducting the interview by the principal investigator. Descriptive content analysis was performed manually by two independent, trained researchers (ANS and PN) to generate categories and themes. These were reviewed by PT and AN to mitigate subjectivity in analysis. The results were reported using the “Consolidated criteria for Reporting Qualitative Research” (COREQ) guideline [9].

### 2.6. Ethics Approval

Ethics approval was obtained from the Institutional Ethics Committee of the MVJ Medical College and Research Hospital, Hoskote, Bengaluru, India, and the Ethics Advisory Group of the International Union Against Tuberculosis and Lung Disease, Paris, France. For the qualitative data, written informed consent for conducting in-depth interviews and audio recording was obtained from all the participants included in the study. In instances where a presumptive TB patient had been missed or diagnosed as TB patient and was not put on treatment, the Senior Treatment Supervisor was immediately informed about it to ensure appropriate follow-up.

## 3. Results

The enablers, challenges, and solutions to improve the ACF activity are described below.

### 3.1. Enablers

**Figure 2:**
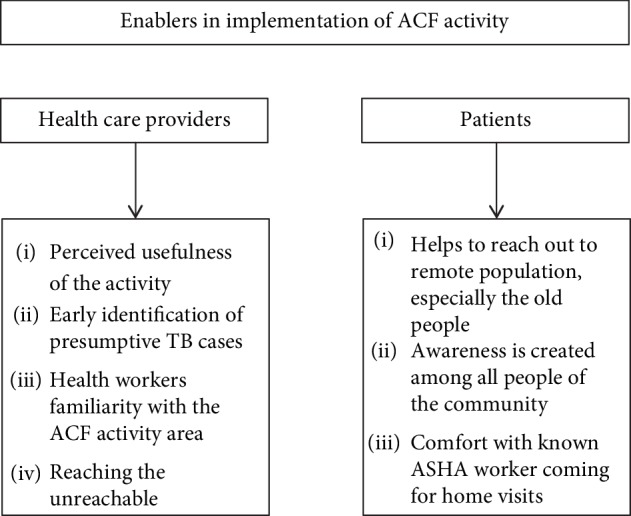
Enablers for ACF activity as perceived by health care providers and patients.

The themes and codes of perceived enablers are depicted in Figure 2. The narrative description of the perceived enablers is given below.

#### 3.1.1. Health Care Provider Related

*(1) Perceived Usefulness of ACF Activity*. All the health care providers interviewed unanimously appreciated the usefulness of ACF activity in the community. Health care providers were of the opinion that this activity increased the awareness about TB in the community, especially on the symptoms suggestive of TB. Thus, it improved health seeking behavior of the presumptive TB patients to come forward and get tested.

“*It is the best screening programme for screening TB suspects in the community. And such screening programme is not there for any other disease. TB being a high burden disease it is a very good initiative for screening TB*.” (TBHV)

“*With this survey, most importantly awareness is being created among the people in the community. In this ACF, high risk groups are found out and because of this, high - risk group people are motivated to come for sputum testing. People think that from health department people are coming to tell us and they feel good and they get the test done*.” (Said a STS)

*(2) Early Identification of Presumptive TB Cases*. Most of the health care providers felt that ACF helps in early identification of the presumptive TB cases.

“*My opinion is that it is good thing started by government. It is the best screening programme for screening TB suspects in the community.*” (ASHA)

*(3) Health Worker Familiarity with the ACF Activity Area*. Health care workers felt that inclusion of community volunteers reduced the potential problems in the community as these volunteers were familiar with the people and geographical boundaries of the allotted area for ACF activity.

“*ASHA workers don't face much problem because usually they will be allotted their area only for survey where people will be knowing them so they will not have much problem*.” (Said a laboratory technician)

*(4) Reaching the Unreachable*. The health care workers felt that ACF activity has enabled them to reach out and provide care to those in hard-to-reach areas and those who are not reachable.

“*Because there will be some people who will be unreachable or not reachable, the ACF gave us opportunity to meet them and educate them on symptoms of TB*.” (Said a TBHV)

#### 3.1.2. Patient Related

The narrative description as perceived by the patients is given below.

*(1) Helps to Reach Out to Remote Population, Especially the Old People*. Most of the patients perceived ACF activity as a good initiative in reaching out to the people at their homes especially those who are old and not ambulatory.

Male patient 1 said, “*Home visits by ASHA are good for those people who cannot go to the hospital. It is especially helpful for the old people who have no one to go along with to the hospital.*”

*(2) Awareness Is Created among All People of the Community*. Most of the patients felt that this activity has helped in creating awareness among the public about symptoms of TB. They also perceived that this activity had removed the accessibility barrier to health care and reduced the cost of illness.

“*Really thankful to the ACF activity because of which I went for sputum testing and was diagnosed early and immediately started on treatment. Otherwise I would have neglected and not got test done and probably my condition would have become complicated.*” (Said by male patient 3)

*(3) Comfort with Known ASHA Worker Coming for Home Visits*. The patients felt very comfortable with the activity as the ASHA, who is from the same area, was involved in screening. Involvement of ASHA helped to generate trust in this activity.

“*I am very comfortable with ASHA worker because I know her from before as she is from our area only.*” (Said by female patient 3)

### 3.2. Challenges

**Figure 3:**
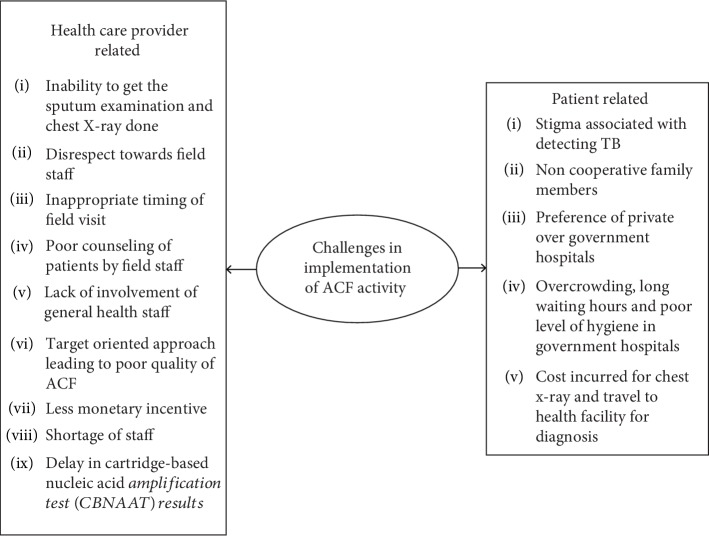
Challenges in implementation of ACF activity.

The themes and codes of challenges perceived by the participants are depicted in Figure 3. The narrative description of the perceived challenges is given below.

#### 3.2.1. Health Care Provider Related

*(1) Inability to Get the Sputum Examination and Chest X-Ray Done*. Health care providers faced major challenge of getting morning sputum sample and chest X-ray done among the identified presumptive TB patients due to various reasons as patients had various misconceptions and were afraid due to stigma associated with TB.

One of the lab technicians mentioned, “*There may be problem because some people refuse to give the sample. Most of the times, people who have symptoms refuse to give the sample because they are scared of being diagnosed as having TB due to the stigma associated with TB. Appropriate counselling has to be given*.”

*(2) Disrespect towards the Field Staff in Community*. Indifferent attitude of the community towards the health care workers due to stigma associated with tuberculosis was another common challenge faced in the community. The field workers also stated that doctors disrespect them in front of patients, and this may lead to the patients not respecting them.

“*And sometimes if patients come late, they scold us in front of the patients. We should not be scolded in front of the patients then they will not respect us. Next time when we go to their house, they feel we are juniors and won't respond*.” (ASHA told)

“*Many times, people don't cooperate. People get irritated if the ASHA workers go to their house. They just don't talk properly and try to send them away by saying that they don't have any problem.*” (As told by one TBHV)

“*People don't co-operate. they are very hesitant to reveal to us. Before only they ask us who are you and why you have come and once they come to know that we are asking something about TB they don't treat us properly and they send us even before we ask them anything.*”(As told by one TBHV)

*(3) Inappropriate Timing of Field Visit*. Field supervisors mentioned that the field workers should visit the houses as per the convenience of the community. However, the field teams do not follow the said timings leading to inability to interact with the community.

“*Usually we should go when people will be at home, but health workers, volunteers' don't follow time. If we tell them to go at 8 am, these staff delay and go by 9 am or 9.30 am. We tell them to either go in the morning or in the evening, then only it will be successful*.” (As told by one of the STS)

“*Almost all come for sputum examination, but they will see their convenience and we have to adjust to their timings. Few people give some reasons and don't turn up. And sometimes they escape by saying that they don't have interest, they have some important work*.” (Said a lab technician)

*(4) Poor Counseling of Patients by Field Staff*. The supervisors were of the opinion that counseling of patients is a weak component in this activity, and thereby, patients are not motivated to come forward for sputum examination.

“*The problem is though we tell some people don't take proper treatment that is because we are lagging behind in giving them proper counseling. So patient counseling as well as family counseling is a very important part and as a supervisor, I feel that counseling component should be strengthened.*” (Said a TBHV)

*(5) Lack of Involvement of General Health Staff*. The NTP staff cited that ACF activity is a huge task and needs involvement of general health staff. It is essential for general health staff to understand the importance of this activity.

“*RNTCP progamme means they feel that only RNTCP staff should do and other departments are not involved. As this is a big survey and just TBHV, STS, STLS can't monitor everything. There should be involvement of all departments. All sectors should be involved in microplanning similar to pulse polio programme.*” (Said one of the STS)

*(6) Target-Oriented Approach Leads to Poor Quality of ACF*. Another important challenge they perceived was ACF being a target-oriented approach; there were possibilities of quality being compromised. Poor quality of sputum brought for lab investigation was common, and this might be due to lack of training of ASHA in sputum collection.

“*It is better to get samples of symptomatic patients. Sometimes the ASHA workers get samples of everyone to complete the target. And sometimes they get saliva instead of sputum to achieve their target. They bring inadequate quantity of sputum.*” (Said by a lab technician)

*(7) Less Monetary Incentive*. Less monetary incentive for ACF activity is one of the major hurdles as perceived by ASHA.

One of the ASHA workers felt and said, “*We don't get our salary on time. 70 Rs incentive is too less for us so they should increase.*”

*(8) Shortage of Staff*. Field workers felt that there is lack of staff in the health facilities. Thus, those patients referred from field have to wait for long hours in the health facilities for doctor consultation and investigation.

One of the ASHA workers said, “*I feel there should be some more doctors appointed during the ACF period so that there will be lesser waiting period and more laboratory technicians also should be appointed so that when the patients go for sputum examination also there should be lesser waiting time.*”

*(9) Delay in Cartridge-Based Nucleic Acid Amplification Test (CBNAAT) Results*. Another challenge faced by the field staff was the delay in getting the CBNAAT results which takes more than 1 week to come.

“*CB NAAT result takes very long time to come so there should be some facility to get the result early. Sputum examination and chest x-rays usually people get it done on time but only this CB NAAT takes more time.*” (One of the ASHA workers said)

#### 3.2.2. Patient Related

*(1) Stigma Associated with TB*. One of the patients felt that the ACF activity is disturbing as the health workers visit their residence. The stigma about TB made people uncomfortable during house visits by health workers.

“*Many times, we don't like them because the neighbours and all will loom at us with suspicion. We believe that if someone gets TB, then they must have done some sin in their last birth because of which God has punished them and they have got TB*.” (As told by female patient 3)

*(2) Noncooperative Family Members*. One of the barriers perceived by the patients in going for sputum examination was noncooperative family members.

“*My husband is not at all supportive, he drinks a lot and whatever I get in my daily wage that money he takes from me and spends on drinking. My husband and I both have cough with sputum, fever, loss of appetite and symptoms of TB as told to me by the asha worker who had come to my house.*” (Said female patient 1)

*(3) Preference of Private Hospitals due to Overcrowding, Long Waiting Hours, and Poor Level of Hygiene at Government Hospitals*. Another barrier which patients perceived was that they preferred going to private hospitals compared to government hospitals because of long waiting time and overcrowding at the government hospitals.

“*I don't like going to Government hospital for anything. I have no financial problem. So, if anything happens, we go to private hospital only. In government hospitals they don't maintain proper cleanliness and if we go there, we are more prone to get infected from other patients as it is always crowded*.” (As said by male patient 2)

*(4) Cost Incurred for Chest X-Ray and Travel to Health Facility for Diagnosis*. One of the problems which patients faced was out-of-pocket expenditure incurred for chest X-ray and travel to health facilities.

“*I was told that the chest x-ray will be done free of cost but when I went get chest x ray done in a medical college, I had to pay for the chest x ray and I had to spend Rs 120 for auto and also missed my daily wage money on that day*.” (Said female patient 3)

### 3.3. Solutions

**Figure 4:**
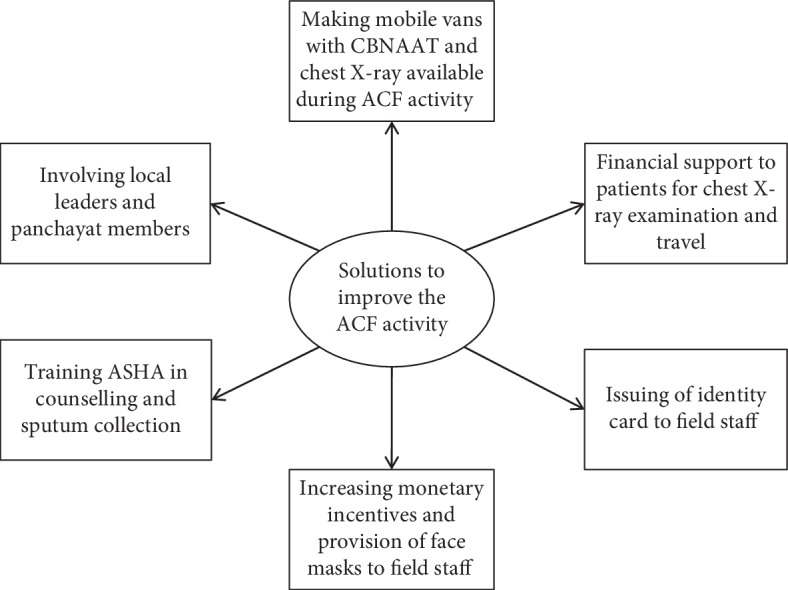
Solutions to improve ACF activity as perceived by health care providers and patients.

The narrative description of the solutions is given below. The themes and codes of the solutions suggested by the participants are depicted in Figure 4.

#### 3.3.1. Health Care Provider Related

*(1) Making Mobile Vans with CBNAAT and Chest X-Ray Available during ACF Activity*. The field staff recommended the mobile vans with CBNAAT and chest X-ray available in the field during ACF activity. They felt this would limit the attrition during diagnosis and also improve the yield of the activity.

“*My suggestion is to install some mobile CBNAAT machines during ACF activity because some patients will oppose going to the health centre*.” (Told by one TBHV)

*(2) Involving Local Leaders and Panchayat Members*. The health workers strongly felt the need for involvement of local leaders and panchayat to motivate the people to participate in ACF activity and come forward for screening.

“*So local leaders and people of the community also should be involved in motivating the people of the community because people will be closer and more comfortable with them.*” (TBHV)

*(3) Training ASHA in Counseling and Sputum Collection*. Training of ASHA in counseling the presumptive TB cases undergo diagnostic tests and sputum collection was one of the solutions proposed to ensure effective ACF activity.

“*The main problem is sometimes they don't get proper quantity and most times they get saliva and put pressure on us to do the test and if I do test then if the sample is not good, we have to do smear, chemicals will be wasted, cups waste and our time will be wasted. So, ASHA workers have to be trained more about how to collect sputum.*” (LT)

*(4) Increasing Monetary Incentives and Provision of Face Masks to Field Staff*. ASHA workers felt that government should provide them with face masks which will help in protecting themselves from infection. One more suggestion which they gave is that their incentives should be increased so that they can work with motivation.

“*I think government should provide us with face masks for our safety because we will be going to TB suspects house. Our incentive is only 70rs per head which is less so that should be increased.*” (ASHA)

*(5) Issuing of Identity Card to Field Staff*. The ASHA workers complained that people do not cooperate with them and do not behave properly and they do not believe that they have come from government for the survey. So the workers suggested that the provision of identity card to the field staff can improve trust among the people.

One of the ASHA workers said, “*from government we should be issued with an id card so that people believe us that we come from government and they cooperate with us. In our area people recognize us so they will give us information but usually they post us to different areas so it is better if they issue an id card.*”

*(6) Financial Support to Patients for X-Ray Chest Examination and Travel*. Few patients suggested there should be provision of financial support to the patients to take care of out-of-pocket expenditure.

“*Some people can't go to give the sample because they won't have the money to take auto and go to the centre. Government can make some arrangements for their travel or someone can come and take the second sample and also come and give them the result of sputum test done.*” (As said by male patient 3)

## 4. Discussion

This is the first study from India to explore the enablers and challenges in ACF implementation as perceived by the various stakeholders and patients using qualitative approach. The findings will help in planning effective ACF implementation hence further.

Most of the health care providers and patients felt that the ACF activity is beneficial as it helps to reach the unreached and helps in early identification of TB patients. Also, people acknowledge the collateral benefits of ACF in terms of improving awareness about TB disease among the general public. However, health care workers faced some challenges like inability to convince people to get sputum test done, indifferent attitude of community due to stigma regarding TB, inadequate training of ASHA workers, shortage of staff during ACF, and delay in getting CBNAAT. Lack of proper counseling by ASHA workers, stigma associated with TB, and shortage of staff in government hospitals were noticed as challenges by both health care providers and patients. The field staff recommended the installation of mobile CBNAAT machine, involvement of general health staff, and training of ASHA in counseling to improve the efficiency of ACF. The field staff and the patients recommended in common that some financial assistance to be provided to the patients for chest X-ray and travel to health facility.

Misconceptions and stigma about tuberculosis in community were a major barrier in identification of presumptive TB patients. Also, the ACF activity was conducted during the working hours and many individuals were out of house during activity. Thus, the health care providers were not able to interview each and every individual in the community. Studies in the past have shown that this compromises the yield of TB cases during ACF [10]. As suggested conducting the activity at the evening time might be a feasible option to ensure individual interviews for elicitation of symptoms.

As suggested by Gilson et al. [11] in the late 1980s, community health workers' services need to be integrated into the health system to maximize their potential and ensure sustainability; however, providing this extension of service to marginalized communities requires careful assessment of additional costs versus benefits. As such, the scalability and cost-effectiveness of community and home screening for TB require further evaluation [12–14].

A study done in South Africa reported a combination of individual (lack of TB knowledge), social (TB stigma), and structural factors (distance, time, and lack of money for transportation to the clinic) impacting their ability to recognize and seek care for TB in a timely manner [15].

A study done in Cambodia [16] reported indirect costs hindering prompt uptake of and adherence to treatment. Long distances to health facilities and transportation challenges aggravated this situation. For those working in the informal sector, where wages are low and work is not steady, the requirement to attend daily facility-based DOTS placed them at risk of loss of livelihood.

Convincing the identified presumptive TB cases to follow up for sputum examination and chest X-ray was a major challenge as perceived by the program staff. Sputum examination and chest X-ray may not be a priority for the patients, and thus, the uptake might be poor. This was in spite of providing all the diagnostic services free of cost. However, studies in the past suggested that in many cases, “free tuberculosis diagnosis and treatment are not enough” [17] given the multitude of costs [18] that TB patients face related to transportation [19] and lost income [20], productivity [21], and time [22]. For those working in the informal sector, where wages are low and work is not steady, the requirement to attend health facilities for diagnosis of TB placed them at risk of loss of livelihood. Thus, improving the access to the diagnostic services by taking into consideration patients' income and wage loss is the mainstay to ensure better uptake of diagnostic tests.

Though there are provisions made for sputum collection and transport for sputum smear microscopy under ACF activity, this might not prevent patient from travelling to health facility as they need to get the chest X-ray done. There were suggestions to provide financial support to the patients against the cost incurred for chest X-ray examination and travel charges. Similar observations were reported in a study from South Africa [15] wherein they suggested the provision of incentives or financial support for medical and nonmedical direct expenses.

The ACF activity was considered NTP activity and thereby received little cooperation from the general health staff. Long waiting hours, poor hygiene at the public health facilities, and thus preference for private health facilities were cited as barriers by patients to avail TB diagnostic services. As suggested by Prasad et al. [10] in the late 1980s, there is need for strengthening the public health system to complement the efforts put during house-to-house identification of presumptive TB patient.

The study has a few strengths. First, we interviewed the health staff at various levels including the general health staff involved in ACF activity. Thereby, the study has been able to capture the varied perceptions. Second, the study reflects the realities in the field. It thus holds implications for the program. Third, the investigator was not involved in program implementation, and thereby, social desirability bias may have been avoided.

The study has few limitations. First, for understanding the perspectives and challenges from the patients, we could interview only eight patients, four among those who completed the diagnosis to treatment cascade and four among those who did not. Thus, we might not have reached the saturation. Second, translation of findings from local language (Kannada) to English could have contributed to distortion of meaning. However, this was abetted through the principal investigator who is fluent in both the languages and through regular discussions with the people who were interviewed to clarify intended meaning. Third, the study results might not be generalized as few of the challenges might be due to local study setting.

## 5. Conclusion

The study offers important insights that will be useful to the program managers and aid effective program implementation. Refusal for diagnosis, high costs, and fear of stigma were identified as leading barriers for adherence in ACF. The health system challenges in conduct of ACF need to be addressed by training the health staff involved in activity and also improving the access to TB diagnostics.

## Data Availability

The data (interview notes) used to support the findings of the study are available with Dr. Amrita N Shamanewadi under license and so cannot be made freely available. Requests for access to these data should be made to Dr. Amrita N Shamanewadi with contact details 9449229007.

## Supplementary Materials

Supplementary Materials COREQ (COnsolidated criteria for REporting Qualitative research) Checklist.
